# Promoting Physical Activity Habits after Completing Secondary School: Does the Age Matter?

**DOI:** 10.3390/ijerph192114160

**Published:** 2022-10-29

**Authors:** María Huertas González-Serrano, Rómulo Jacobo González-García, Ana Gómez-Tafalla, Ignacio Refoyo Román, Fernando García-Pascual, Ferran Calabuig

**Affiliations:** 1 Department of Physical Education and Sports, Faculty of Physical Activity and Sport Sciences, Universitat de València, 46010 Valencia, Spain; 2 Campus Capacitas, Universidad Católica de Valencia, 46110 Valencia, Spain; 3 Departamento de Deportes, Universidad Politécnica de Madrid, 28040 Madrid, Spain

**Keywords:** intention to be physically active, physical exercise identity, physical self-concept, theory of planned behaviour, physical education, secondary education

## Abstract

Physical inactivity is one of the most important problems in our society, especially during adolescence. During this stage of schooling, students practice physical activity in physical education classes, but after they finish school, many of them stop practicing it. This research aims to determine which variables predict the intention to practice physical activity after finishing secondary school and to discover the effect that age during adolescence has on these predictive variables. A questionnaire was administered to a sample of secondary school students. The results show that physical exercise identity influences the three antecedents of this theory, while the attitude towards the behaviour (β = 0.13; *p* < 0.05), perceived behavioural control (β = 0.46; *p* < 0.05), and physical self-concept (β = 0.41; *p* < 0.05), have a statistically significant influence on the intention to be physically active. Moreover, when the age is lower, is more important to develop a positive perceived behavioural control and physical exercise identity. Moreover, for younger male adolescents instead of the physical exercise itself, identity seems more important in developing a positive attitude towards behaviour. Likewise, in young female adolescents a low level of support from their close environment (subjective norm) leads to dropping out. The research concludes with the importance of including these variables in physical education classes to promote physical activity practice after completion of secondary school. Some practical implications are presented.

## 1. Introduction

Physical inactivity is considered to be the fourth largest cause of death worldwide [1,2]. The absence of physical activity practice can lead to non-communicable diseases (NCDs) such as heart disease, hypertension, overweight, obesity, diabetes and increases the risk of some types of cancers and mental diseases [3]. Physical inactivity causes 5.2 million deaths, in addition to 6–10% of major noncommunicable diseases annually worldwide [4]. For instance, the rate of obesity in children and adolescents has increased tenfold in the last four decades, with sedentary lifestyles being one of its main causes [5]. According to the World Health Organization [6], 80% of adolescents (aged 13–17), and 23% of adults (18 or more years) do not meet the moderate-to-vigorous physical activity guidelines of 60 min per day. These data show how decreases in physical activity occur during the adolescence stage [7] and continue later, in adulthood [8].

Thus, age during the adolescence period seems to be an important factor that could affect future physical activity practice. Concerning gender differences, many studies identify girls as being less active than boys during adolescence [9,10]. A physically inactive lifestyle from youth is associated with an unfavorable cardiometabolic risk profile in adulthood [11]. Sedentary behavior replaces light activity throughout adolescence, and is associated with an increased risk of depressive symptoms at age 18 [12]. Moreover, adolescence is an early period of life that is very important for promoting physical activity and reducing the incidence of depressive symptoms throughout adulthood [13]. In this vein, O’Donoghue et al. [14] conducted a systematic review to identify associations between sedentary behavior and age in adults aged 18 to 65 years. The results showed that 14 of the 20 studies found a positive relationship between age and sedentary behaviour (the older the person, the more sedentary).

Moreover, there is a growing lack of interest in physical education lessons during this stage [15]. This fact is especially worrying because the years of schooling represent a critical period in the development of habits of physical and sports practice and their subsequent transfer to adult life [16]. In addition, the regular practice of physical and sports activity by adolescents is related to lower consumption of harmful substances for health and higher academic performance [17].

According to the recommendations [6], adolescents should do at least 60 min of moderate to vigorous-intensity physical activity daily. Additionally, at least three times per week, activities that strengthen muscle and bone should be carried out. The practice of this sort of physical activity can prevent depression and mental health disorders [18], cardiovascular disease [19], metabolic disease [20], and some cancers [21]. Furthermore, physical activity practice is also related to the improvement of mental cognition [22], and academic performance [23].

Among all institutions, schools have the strongest influence on the young population [24]. Therefore, Physical Education (PE) classes should be aimed at solving this problem so that PE objectives include teaching students to adopt regular physical activity habits during their free time [25]. Thus, schools play a substantial role in combating the worldwide prevalence of physical inactivity and promoting physical activity during this stage [26]. As the number of PE classes during secondary school is limited, however, it is of vital importance to develop enriching experiences during them [27]. Therefore, it is necessary that PE classes are of good quality, and that a related culture is fostered that contributes both to knowledge and to the development of healthy habits and lifestyles [28]. Thus, PE teachers play a key role in promoting students’ acquisition of an active lifestyle [25].

Hence, the importance of physical education (PE), which is a compulsory subject for all the years in which children exhibit higher levels of susceptibility to dropping physical activity, makes PE an attractive field for analysing variables related to physical and sports practice, such as the intention to be physically active [29]. Research on the correlations and determinants of physical activity has accelerated over the past two decades [30]. However, more research is still needed on the factors associated with persistence in physical exercise [31], because although some factors have been recognized as important concerning physical activity practice, the psychological determinants are still uncertain [32]. In this vein, Loh et al. [33] highlighted that it is essential to discover the determinants of physical activity among adolescents, particularly beyond intrapersonal influences, to develop effective public health interventions to ensure long-term health benefits.

Therefore, the interest of this study lies in the fact that very few studies have analysed the indirect effect of physical exercise identity on the intention to engage in physical sports practice through the TPB variables. To the best of our knowledge, no previous studies have introduced both physical exercise identity and physical self-concept within the TPB to explain the intention to practice physical activity after finishing secondary school using structural equation models. Besides, no previous studies have been found that combine a symmetric (Structural Equations Modelling) and asymmetric (fuzzy set Qualitative Comparative Analysis) approach to analyse IPA. Researchers recommended the combination of these two methods to provide a better understanding and more insights into the relationships between variables [34]. Hence, the main objectives of this study are (1) to determine the predictive variables of the intention to practice physical activity after finishing secondary school, and (2) to analyse the effects that age during adolescence has on these variables in general, and through a gender lens.

### Theoretical Framework and Hypothesis Proposals

Regarding the determinants of physical sports practice, it is worth noting that the Theory of Planned Behaviour (TPB) [35] has recently become one of the most common theories used to understand physical activity in young people as well as in numerous populations [36]. These studies indicate that people who have a favourable attitude towards physical activity (attitude towards behaviour), who perceive that those significant to them expect them to perform such activities (subjective norm), and who express strong feelings of control over their activities (perceived behavioural control) are more likely to state strong intentions to engage in physical activity. Therefore, the following hypotheses are proposed:

**Hypothesis** **1** **(H1).** *The attitude towards behaviour directly and positivelyaffects the intention to be physically active after finishing secondary school*.

**Hypothesis** **2** **(H2).** *Perceived behavioural control directly and positively* *affects the intention to be physically active after finishing secondary school.*

**Hypothesis** **3** **(H3).** *The subjective norm directly and positively* *affects the intention to be physically active after finishing secondary school.*

However, the TPB allows the consideration of additional predictors if they capture a significant portion of the behavioural intention after the current variables of the theory have been taken into consideration [35]. Therefore, Conner [37] added new variables to the theory. Self-concept is especially important in adolescence, since everyday feelings are important in personal development, which is subjective and varies according to external factors as well as new life contexts [38]. Thus, another of the determinants of physical sports practice that has been the object of study is the physical self-concept (PSC), which is the positive opinion and feelings (happiness, satisfaction, pride, and confidence) associated with the physical dimension. Several studies in recent decades have shown direct relationships between the physical self-concept and physical sports practice [39,40]. Thus, the following hypothesis is proposed:

**Hypothesis** **4** **(H4).** *The physical self-concept directly and positively affects the intention to be physically active after finishing secondary school*.

Physical exercise identity (PEI) is another frequently studied variable. In fact, a meta-analysis found that adding this variable to the TPB could explain between 2% and 6% more of the variance in the intention to perform different health-related behaviours [41]. Physical exercise identity is defined as “the degree to which an individual identifies with the athlete role and looks to others for acknowledgment of that role” [42], p. 237. Identity theory proposes that identities and behaviours are congruent, so a strong perceived fit between identity and a particular behaviour is related to a strong intention to represent that behaviour, making the realization of the behaviour more likely. Therefore, in the literature on social and sports psychology, physical exercise identity has received widespread attention and has proven to be important for health and fitness outcomes worldwide [43]. In this vein, Erdvik et al. [44] highlighted that physical activity identity contributes to making individuals more likely to develop positive physical activity intentions. In addition, some authors [45] note that individuals with a strong physical exercise identity are more likely to play sports than individuals with a weak physical exercise identity. Strachan et al. [46] suggested that physical exercise identity and self-regulation predict intentions for future physical activity practice. Besides, the relationship between PEI and attitude towards behaviour and perceived behavioural control has proven to be positive and significant [47]. Thus, PEIs exert their influence through IPA within the Theory of Planned Behaviour indirectly, through their three antecedents. Moreover, PEI has a relationship with the physical self-concept [48], because both variables are related to physical self-perceptions. Thus, the following hypothesis is proposed:

**Hypothesis** **5** **(H5).** *The physical exercise identity is correlated with the physical self-concept*.

**Figure 1:**
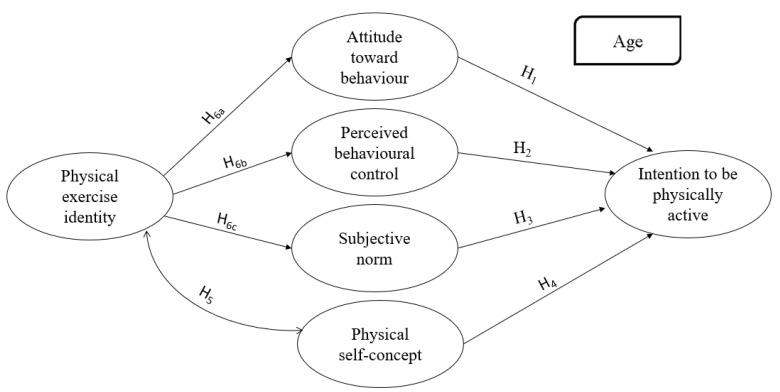
Hypothetical model of the intention to be physically active after finishing secondary school and associated variables with the TPB, including physical exercise identity, and physical self-concept.

Other authors [37] examined the effects of people’s self-identity on their intention to engage in physical sport, showing their results not to be direct but indirect effects of self-identity on behavioural intentions, mainly through the TPB antecedents [49]. Few other studies have examined the role of self-identity in the antecedents of TPB in human behavioural intentions [50]. For this reason, the following hypotheses are proposed (see Figure 1):

**Hypothesis** **6a** **(H_6a_).** *Physical exercise identity positively affects the attitude towards behaviour directly and positively.*

**Hypothesis** **6b** **(H_6b_).** *Physical exercise identity positively affects perceived behavioural control directly and positively*.

**Hypothesis** **6c** **(H_6c_).** *Physical exercise identity positively affects the subjective norm directly and positively*.

## 2. Materials and Methods

### 2.1. Participants

The sample comprised 252 students who were studying at a high school, whose ages ranged between 16 and 19 years old (M = 16.36; SD = 0.62). A non-probabilistic intentional or convenience sampling was used for collecting the data ensuring the representativeness of the data collected (95% interval confidence, with 5% of error). The number of male students is similar to that of female students, with a slightly higher number of boys (55.60%) than girls (44.40%). For the analysis of the sample according to the courses, of the 286 students that comprised the sample, 89 (30.90%) were in the 3rd year of Secondary School, 89 (30.90%) were in the 4th year of Secondary School, 83 (28.50%) were in the 1st year of A levels, and 25 (9.80%) were in the 2nd year of A levels, in which the subject of Physical Education is not compulsory. In the following Table 1, more detailed characteristics of the sample related to their sports habits and the sport habits of their environment are presented.

### 2.2. Instrument

For the collection of information, a questionnaire composed of 40 items was created, divided into different areas, taking the following scales as a reference:

(a) *Intention to be Physically Active*: The Spanish-validated version [51] of the Intention to be Physically Active Questionnaire [52] was used. This scale is composed of five items that measure the personal intention to be physically active at the end of schooling (e.g., “After graduation, I would like to be physically active”). The items are preceded by “Indicate the degree of compliance with the following issues”. A five-point Likert scale was used for item responses, where 1 means totally agree and 5 totally disagree. The scale showed a Cronbach’s alpha of 0.79 in this study.

(b) *Self-Concept*: The Abbreviated Physical-Self Concept Questionnaire (CAF-A) [53] was used. This scale is composed of the following eight items, two for each scale: physical ability (e.g., “I am clumsy doing physical activity”), physical condition (e.g., “I have a lot of physical endurance”), physical attractiveness (e.g., “I am happy with my physical body image”) and strength (e.g., “I am a strong person”). The items are preceded by “Answer the following items, where 1 means false and 5 true”. The Cronbach’s alpha of this scale for the sample of this study was 0.72.

(c) *Theory of Planned Behaviour*: The questionnaire on planned behaviour [54] was used. Three dimensions were used: (1) subjective norms, composed of four items (e.g., “Most people important to me think I should exercise at least six times in the next two weeks”), and (2) perceived behaviour, composed of five items (e.g., “If I wanted to, I could exercise at least six times in a week the next two weeks”) and (3) attitude towards behaviour, composed of seven items starting with “For me to exercise at least six times a week times in the next two weeks would be...”, considering in each item a pair of opposing adjectives (e.g., “very bad—good”, “not important—very important”., etc.), which were answered on a Likert scale ranging from 1 for the most negative attitude to 7 for the most positive attitude. For the other items, a Likert scale of seven points was used, with 1 indicating total disagreement and 7 indicating total agreement. The reliability values obtained for the sample in this study were α = 0.82 for the control of perceived behaviour, α = 0.87 for the attitude toward the behaviour, and α = 0.84 for the subjective norm.

(d) *Exercise Identity Scale*: The Spanish translation of the nine-item Exercise Identity Scale (EIS-S)[55] was used to measure students’ identification with the role of physical activity (e.g., “Physical exercise is a central factor of my self-concept”). The items were preceded by “Indicate the degree of agreement with the following issues” and answered on a 5-point Likert scale where one indicates complete disagreement and five complete agreement. Cronbach’s alpha on this scale for the sample in this study was 0.91.

The questionnaire also included various socio-demographic data as well as a short introduction explaining how to fill out the questionnaire. It emphasized the anonymity of the data and stated that they will be used solely for academic purposes.

### 2.3. Common Method Bias

To ensure that there is no common method bias, the language of the questionnaire items was maintained as simple as possible. Double-barrelled questions were avoided, and variables were explained before their measurement items to generate psychological separation [56]. Moreover, one post hoc test was conducted to assess the common method bias, namely Harman’s one-factor [56].

Harman’s single factor test was performed to verify whether the variance explained by all 33 items under one single factor is below 50% of the explained variance or not. In this case, the explained variance was 30.62%, which is below the threshold limit [56]. In that way, it could be ensured that the study is not affected by the common method biases.

### 2.4. Procedure

Before starting the data collection, the high school was first contacted, the high school headmaster was asked for permission to conduct the research, and the questionnaire was sent to him for review and consent. Then, the Ethical approval was obtained from the Ethics and Human Research Committee of the University of Valencia. Informed consent was requested from students’ parents. Most of the parents filled out the form. The guidelines of the 1964 Declaration of Helsinki and its subsequent updates were followed [57].

We used purposive sampling for convenience; that is, we tried to administer the questionnaires to as many students as possible in the 3rd and 4th grades and A levels of a subsidized school in one city in Spain. These students were chosen because they are close to the end of their compulsory schooling (3rd and 4th grades) or their schooling period (A levels) in high school and the end of the subject of Physical Education, and they may readily think about their future sports practice after finishing secondary school. Data was collected during the last two weeks in April of the academic year 2018/2019.

The time required to answer the questionnaire was approximately 10 min, with no incidents due to delivery delays.

### 2.5. Data Analysis

The data obtained were subjected to various statistical analyses using the SPSS version 23 statistical package and the EQS 6.3 software. First, a descriptive (mean and standard deviation) and reliability (Cronbach’s alpha) analysis was carried out through the SPSS program, as well as a correlational analysis between the variables under study (Pearson correlation).

Subsequently, and once it was verified that the necessary assumptions were met, two models of structural equations were carried out with the program EQS 6.2. For all models, an estimator of maximum likelihood was used with the robust method given the absence of multivariate normality (Mardia coefficient = 30.043). The following indicators were used to evaluate the goodness of fit: Chi-square (*X*^2^), Satorra–Bentler scaled Chi-square (*S-BX*^2^); *S-BX*^2^ divided by the degrees of freedom, the root mean square error of approximation (*RMSEA*), the non-normed fit index (*NNFI*) and the comparative fit index (*CFI*).

Besides, the Qualitative Comparative Analysis methodology was used. Fuzzy-set qualitative comparative analysis (fsQCA) shows the conjunctions of all logically possible configurations (combination of conditions) to achieve a specific outcome [58]. One of the main characteristics of this methodology is equifinality, which means, it presents different paths to achieve a specific outcome [59]. This methodology is based on the logic that a specific condition on an outcome depends on the combination of different conditions.

First of all, all missed data were deleted, and raw data responses were transformed into fuzzy-set responses. To estimate all the conditions the mean of the items was calculated. Next, all conditions were recalibrated with values between 0 and 1. For the recalibration of the conditions with more than two values (continuous variables), it is required to select three thresholds. The first one (0) refers to that observation with this value is fully outside the set (low levels), the second one (0.50) represents a median point (intermediate levels), and the last value (1) considers the observation to be fully inside the set (high levels). Woodside [60] recommends that the following three thresholds must be percentile 10 (low levels), 50 (intermediate levels), and 90 (high levels). All the continuous variables (conditions) were recalibrated using these three thresholds.

After that, necessary and sufficient conditions tests were performed. On the one hand, the condition is necessary when it must be always present or absent for achieving a particular outcome (high or low levels of IPA). Ragin [61] suggests that for a condition to be considered necessary, the consistency value must be superior to 0.90. On the other hand, a sufficient condition represents the combinations of conditions (configurations) that can achieve a specific outcome. fsQCA (University of California, Irvine, CA, USA) is composed of two stages to perform sufficient conditions [58]. Firstly, a truth table algorithm converts the fuzzy-set membership scores into a truth table. Solutions should be ordered by their raw consistency in descending sense. The consistency threshold should be selected by observing large breaks in consistency values in this truth table [62]. Concerning the frequency threshold, the cut-off value used to select the number of cases that must be connected with a configuration is to be further considered [63].

Secondly, fsQCA presents three possible solutions: complex, parsimonious, and intermediate. Intermediate solution has been used in this research as suggested by Ragin (2008). The parsimonious solution was used to detect the core and peripheral conditions. Core elements indicate a robust causal relationship with the specific outcome, and peripheral elements indicate a softer relationship with the outcome [64].

The SPSS (Statistical Package for the Social Sciences, Version 24) (IBM Corp, Armonk, NY, USA), and the fsQCA 2.0 software were used. SPSS was used for the descriptive analyses of the variables (mean, standard deviation, scale averages, minimum and maximum values, and percentiles), and the correlation analysis. Finally, fsQCA 2.0 was used to perform the other analyses.

## 3. Results

In this section the statistical analyses to respond to the objectives and hypotheses are presented. Firstly, the descriptive, reliability, and correlational analyses are presented, and finally the results of structural equation models to predict IPA.

### 3.1. Analysis of Reliability, Averages, and Correlation between Variables

Table 2 presents the means of the different variables related to physical activity practice. It is important to highlight the variables of perceived behavioural control ($\bar{X}$ = 4.46; *DT* = 0.71) and intention to be physically active ($\bar{X}$ = 4.26; *DT* = 0.78) as the ones with the highest means, and the variables of physical self-concept ($\bar{X}$ = 3.61; *DT* = 0.76) and subjective norm ($\bar{X}$ = 3.76; *DT* = 1.00) as the ones with the lowest mean scores (see Table 1).

Subsequently, an analysis of the correlations between the variables composing the study model was carried out. As shown in Table 2, all independent variables are significantly correlated with the dependent variable IPA.

Of the independent variables with the highest correlation with the dependent variable, the AI with r = 0.81 (*p* < 0.001) and the PSC with r = 0.50 (*p*< 0.001) should be highlighted. On the other hand, concerning the variables with lower correlation values, the SN (r = 0.31; *p* < 0.001) and the ATB (r = 0.34; *p* < 0.001) should be highlighted, as shown in Table 1. However, age did not present any statistically significant correlation with the other variables.

### 3.2. Structural Equation Model

The structural equation analysis was performed by adding two new variables to the TPB variables (ATB, PBC, and SN): physical self-concept and physical exercise identity. A model was proposed in which attitude towards behaviour, perceived behavioural control, subjective norm, and physical self-concept directly influenced intention, and identity had an indirect influence through the other variables.

When testing the second structural equation model with the hypothetical relationships that were established between the TPB variables and PSC and PEI, the results of the model also presented a good fit, with the following values: χ (247.54 gl = 127 *p* < 0.001); *S-B χ/gl* = (213.16/127 = 1.70); *RMSEA* = 0.05 (0.03–0.08); *NNFI* = 0.94, and *CFI* = 0.95. All these values are also recommended in the literature: x^2^/gl below three [65], *RMSEA* with a value below 0.80 [66], and finally, the *NNFI* and *CFI* indices with values above 0.90 [67].

**Figure 2:**
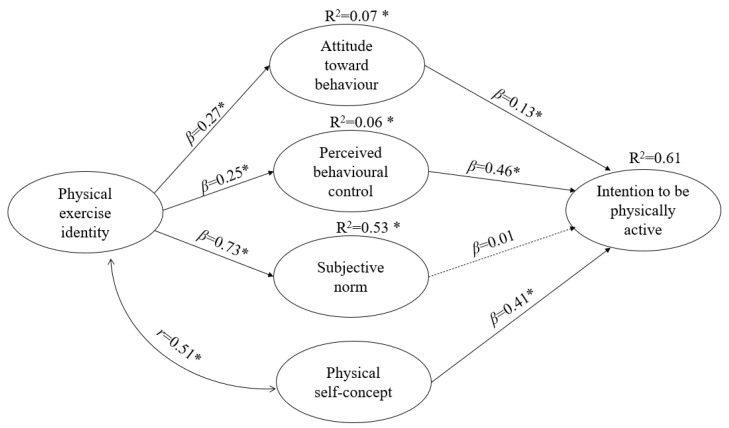
Model of causal relationships influencing students’ IPA after completing secondary school and TPB variables, AI and PSC Note: * *p* < 0.05. Model fit: χ (gl) = 247.54 (127); S-B χ (gl) = 213.16 (127), NNFI = 0.94, CFI = 0.95, RMSEA = 0.06 (0.03–0.08). The discontinuous line indicates a non-significant relationship.

In this model, the dependent factors are ATB, PBC, SN, PSC, and the IPA after completing secondary school. There is only one independent factor that corresponds to the variable PEI. Figure 2 shows that physical exercise positively and directly impacts the three TPB antecedents. PEI influences the ATB and PBC variables, with *β* = 0.27 (*p* < 0.05) and *β* = 0.25 (*p* < 0.05), respectively. Moreover, the greatest path coefficient of the model is the one that relates physical exercise identity to the subjective norm (*β* = 0.73; *p* < 0.05). Thus, hypotheses 5_a_, 5_b_, and 5c are supported. Besides, the model shows that PEI is positive and strongly correlated with PSC (r = 0.51; *p* < 0.05), confirming hypothesis 6. Therefore, the PEI of students who are about to complete their secondary and high school studies is of great importance in their ATB. In addition, there is a medium correlation between PEI and the physical self-concept (r = 0.51; *p* < 0.05).

**Figure 3:**
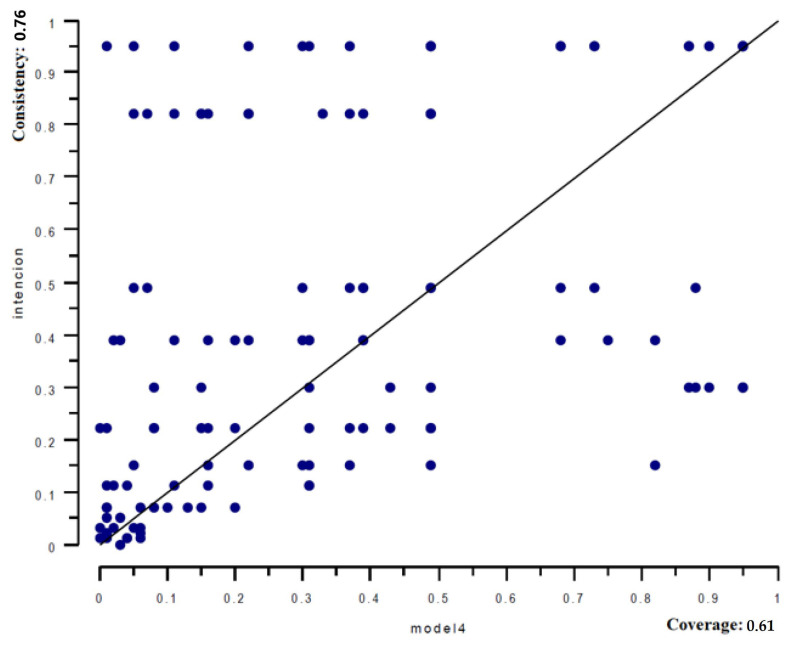
Test of Model 4 in sub-sample 1 using data from sub-sample 2.

On the other hand, regarding the variables directly related to the IPA after finishing secondary school, the variables PBC (β = 0.13; *p* < 0.05), ATB (β = 0.46; *p* < 0.05), and PSC (β = 0.41; *p* < 0.05) were significant. However, the SN variable was not significant, as in the previous model (see Figure 3). In this way, 61% of the variance of the IPA after finishing secondary school was explained through the model (see Figure 2).

### 3.3. Fuzzy-SET Qualitative Comparative Analysis (fzQCA)

Firstly, the descriptive statistics of the variables under study are shown. The means and standard deviations of the variables were calculated. These values can be observed in Table 3 below.

In the case of the gender variable, it was coded as a dummy variable (0 = female; 1 = male). Subsequently, analysis was performed for both high and low (~) levels of intention to be physically active at the end of secondary school. As can be observed in Table 4 for high levels of IPA, high levels of perceived behavioural control turned out to be a necessary variable for such an outcome. This is because the consistency of this variable exceeded 0.90 as recommended by Ragin [61]. 

Likewise, for low levels of IPA, low levels of physical self-concept also proved to be a necessary condition [34]. As in the previous case, the consistency of this variable exceeded 0.90 which is the cut-off point recommended by the literature [61].

For the intermediate solution, it was considered that the presence of all conditions except the age and the gender (that could be present or absent) leads to high levels of IPA. The frequency cut-off in the truth table was set to 3, due to the sample size being larger than 150 cases [64,68]. The consistency cut-off was set to 0.80. The intermediate solution comprised three causal configurations (combinations of conditions) that were able to explain 76% of the cases of intentions to be physically active after completing secondary school (consistency: 0.76; raw coverage: 0.78).

Table 5 shows the three solutions for explaining the cases of high levels of IPA. The notation employed by Fiss [64] was used to present the results. Black circles indicate the presence of a condition, and white circles indicate the absence of a condition. The most important or explanatory configuration for having high levels of IPA was high levels of attitude towards behaviour * high levels of perceived behavioural control * high levels of physical exercise identity (consistency: 0.80; raw coverage: 0.61). The second main configuration was high levels of perceived behavioural control * high levels of physical exercise identity * low levels of age (consistency: 0.82; raw coverage: 0.59). The third and last configuration was high levels of attitude towards behaviour * high levels of perceived behavioural control * low levels of age * to be a male adolescent (consistency: 0.84; raw coverage: 0.31). These three solutions were able to explain 61%, 59%, and 31% of the variance of high levels of IPA.

Consequently, the sufficiency analysis for low levels of IPA was performed. The threshold was established at 0.81, and the frequency cut off at three cases. Four solutions were obtained that were able to explain 70% of the cases of low levels of IPA (consistency: 0.85; coverage: 0.70). The most important configuration for low levels of IPA was high levels of attitude towards behaviour, high levels of perceive behavioural control * low levels of physical exercise identity (consistency: 88; coverage: 0.47). The second main configuration was low levels of physical exercise identity * to be a female adolescent (consistency: 86; coverage: 0.42). The third most explanatory configuration was low levels of attitude towards behaviour*low levels of physical exercise identity * low levels of age (consistency: 89; coverage: 0.41). The last solution was low levels of subjective norm * low levels of age * to be a female adolescent (consistency: 0.83; raw coverage: 0.26). These solutions were able to explain 47%, 42%, 41% and 26%, respectively, of the variance for low levels of IPA.

The predictive validity test was conducted following the recommendations of Papas and Woodside [69]. The procedure was the following: (1) The database was divided into two sub-samples with an equal number of cases; (2) the first sub-sample was used to perform the fsQCA analysis with the same criteria as in the original analysis with the total data sample; (3) the configurations obtained (fuzzy-set models) were taken from the first sub-sample, and they were performed in the holdout sample (second sub-sample); (4) the different models were tested in the holdout sample, using the XY-plot graph; (5) steps 3 and 4 were repeated using the holdout sample to test all the models of the first sub-sample. Next, the consistency and coverage values of the two sub-samples were compared. The consistency and coverage values of the models from sub-sample 1 should be similar to the ones from the holdout samples. If this criterion is met, high predictive validity could be ensured. 

The XY-plot graph from Model 4 was tested in the holdout sample (Figure 3). Models with consistency near 0.80 could be helpful and can help theory advancement [70]. In this case, 0.76 indicates high consistency, while 0.61 indicates the coverage. These values indicate that the data is consistent (76%) with the argument that Model 4 is a subset of IPA that covers 61% of the cases. Thus, this test reveals a high predictive capacity of the solutions obtained.

Lastly, the robustness of the results was evaluated. The test to analyse the changes in the frequency and consistency thresholds was used [71]. The results did not fluctuate considerably from the initial set, although small changes may generate significant changes in the final solution [72]. The results with consistency thresholds of 0.83 were the following: (1) low levels of age * to be a male adolescent * high levels of physical exercise identity * high levels of perceived behavioural control (consistency: 0.88; raw coverage: 0.40); (2) low levels of subjective norm * high levels of perceived behavioural control * high levels of attitude towards behaviour (consistency: 0.86; raw coverage: 0.37); (3) to be a male adolescent * high levels of physical exercise identity * high levels of perceived behavioural control * high levels of attitude towards behaviour (consistency: 0.86; raw coverage: 0.37); (4) low levels of age * high levels of exercise identity * low levels of subjective norm*high levels of perceived behavioural control (consistency: 0.86; raw coverage: 0.36); (5) low levels of age * high levels of physical self-concept * high levels of physical exercise identity * high levels of perceived behavioural control * high levels of attitude towards behaviour (consistency: 0.91; raw coverage: 0.34); (6) low levels of age * to be a male student * high levels of perceived behavioural control * high levels of attitude towards behaviour (consistency: 0.84; raw coverage: 0.31). All the tests confirm the predictive validity and robustness of the results presented.

## 4. Discussion

One of the greatest challenges of physical education is generating habits of physical activity and sports practice outside the school, and above all, ensuring that these habits last throughout the student’s life. However, this objective is difficult to achieve, especially if the variables that influence it are unknown. Hence, the main objectives of this study were to determine the predictive variables of IPA after finishing secondary school and to discover the effect that age during adolescence has on these predictive variables of IPA.

The sample of this study shows that the vast majority of the students practice physical activity as an extracurricular activity. Hence, through physical education classes, these indexes should be maintained, or even increased to enhance physical activity practice during adolescence and adulthood. In this vein, the validity of the TPB [35] for the study of the intention to practice physical activity has been validated for the population of students analysed. The findings of this study show that the variables of attitude towards behaviour and perceived behavioural control can explain the IPA after finishing secondary school. These results are in line with those obtained in a review of the determinants of physical activity practice in adolescents, which concluded that subjective norms had no statistically significant relationship with physical sports practice in most of the studies reviewed [73]. This may be because, as some authors noted [74], participation in physical activity is based more on personal motivation and less on the influence or pressure of others in the environment. In addition, perceived behavioural control was one of the most important variables, which aligns with the findings of previous researchers [49]. Therefore, students should be provided with the necessary skills to be able to practice physical activity outside the PE classroom independently.

Besides, the findings of this study are similar to those of several previous studies [41,75] that also used the TPB and added new variables to find that physical exercise identity was one of the most influential variables in the prediction of IPA. Likewise, our data reveal that exercise identity is an antecedent of all the TPB variables, exerting influence indirectly on the three antecedents of the TPB, as a previous study has shown [49]. However, other authors have only found a relationship between two of them [47]. This means that people who rate their physical exercise identity highly are more likely to form positive attitudes and expectations of control regarding future behavioural engagement in physical sports practice. Therefore, it is necessary to promote motivational programmes for students to improve their physical activity levels and physical exercise identity [76]. Furthermore, encouraging children to see themselves as sportsmen and women can be a strategy to promote physical sports practice during the educational stage [41].

As for the physical self-concept variable, in this case, it also turned out to be a predictor of intention, as some previous studies have already demonstrated [51]. In addition, the correlation found between physical exercise identity and the physical self-concept is in line with Anderson [48] and highlights that by developing specific characteristics, such as the physical self-concept, people could build multiple identities defined by their attributes, such as physical exercise identity. This could be helpful because an individual’s self-perception is thought to influence the ways he or she acts, and the acts in turn influence the perception of the self [77]; this two-way influence can affect physical aspects. For this reason, the results obtained in this study show, as other authors have previously highlighted [28], that in PE classes, beyond the mere practice of physical activity (aspects of physical condition), a culture related to physical activity must be promoted (psychological aspects), which contributes to the knowledge and the development of critical attitudes of personal responsibility and healthy habits and lifestyles.

Concerning age, although the effect of age was not found in the SEM model, in the QCA model it was found to be an important variable. The insignificant effect of age in the SEM model could be because of the characteristics of the sample size. Thus, future longitudinal studies with larger sample size should be developed to further explore the effect of age in this SEM model. The QCA model data showed that the younger the adolescents are, the more important it is that they have a very positive perception of their physical exercise identity and of the control and self-efficacy they feel towards regular physical-sports practice. Moreover, in the case of male adolescents, specifically, instead of perceiving a high physical exercise identity, it is quite important that they have a positive perception of the importance of the regularly physical activity practice. This need to create a positive attitude towards sports practice, a positive attitude towards physical activity practice and a positive exercise identity when adolescents are younger may be due to the fact that as they move through the grades, physical education becomes less and less important while other subjects become more important. This finding is in line with O’Donoghue et al. [14] who found a positive relationship between age and sedentary behavior: the older the person, the more sedentary they are. This is also aligned with Fernández et al. [78] who found that life demands are one of the most important barriers for adolescents deciding not to practice physical activity. In this vein, lack of time, increased workload, and preference for other leisure activities have been also highlighted as hampering factors in adolescents for not practicing physical activity [79]. Thus, it is very important to instill from an early age (first years of secondary education) in adolescents the importance of regular physical sports activity and to provide them with the necessary tools to do so, developing in this way their exercise identity. In this way, adolescents and future adults will be able to better organize their time and establish physical sports practice as a priority in their agendas.

Besides, in the specific case of younger female adolescents, if they perceive that their environment is not supporting them to practice physical activity regularly, they will present low intentions to practice physical activity. Thus, in the case of younger female adolescents, their immediate environment seems to be very important to inhibit or foster their physical activity practice. These findings are in line with Laird et al. [80] who in his meta-analysis noted that parents and friends may have a role in improving physical activity practice in adolescent girls. In the case of female adolescents, independent of their age, if they perceived themselves with a low physical exercise identity, they would develop low intentions to practice physical activity in the future. Thus, enhancing physical exercise identity in general, but specifically in female adolescents is vital to develop the intentions to practice physical activity. This finding is aligned with Bean et al. [81] who highlighted the importance of developing a physical exercise identity in adolescents girls.

Therefore, it is necessary to develop adequate policies to promote these positive attitudes in the upper years of Secondary School and baccalaureate. In addition, providing students with the tools to complete their 60 min of vigorous physical-sports practice three days a week on their own is important. These findings may help to reduce the levels of sedentary lifestyles that increase during adolescence [7]. About the factors that may inhibit it, it seems that younger pupils in Secondary School who have a lower or negative perception of their attitude towards regular physical-sports practice, whose environment is not very supportive of such sporting practice, and who have a low physical self-concept, are more likely to be sedentary when they finish their schooling. Therefore, physical self-concept should be worked on at an early age, as well as days in which families should be involved in physical sports practice with their children.

In general, the data show that it is necessary to intervene jointly on the different variables, and not only in isolation. They also show that the proposals for the promotion of the practice of physical and sporting activity can be more or less effective considering the variables worked on according to age. These findings are in line with Rasoolimanesh et al., [34] who point out that as a complement to the evaluation of the explanatory and predictive power of the SEM model, fsQCA generates a more detailed view of the relationships between variables, thus providing the means to reach better conclusions. 

Therefore, it is worth mentioning the contribution of this study to the existing literature on the variables related to physical activity practice in the stage of adolescence through the analysis of structural equation modelling and Qualitative Comparative Analysis methodology. Specifically, the introduction of two new variables to the TPB, namely, the physical self-concept and physical exercise identity, can explain a large percentage of the variance of the IPA. In addition, the correlation between the physical self-concept and physical exercise identity should be highlighted, as well as the direct influence of physical exercise identity on the three antecedents of the TPB and thus its indirect influence on the IPA. Moreover, although in the case of the SEM model the age did not have a significant effect mediating the relationship between variables (maybe because the sample size), in the QCA model, age played an important role.

Thus, considering the findings of this study, encouraging perceived behavioural control, attitude towards behaviour and physical self-concept would help increase the intention of physical activity practice. However, the promotion of physical exercise identity is also important since this variable exerts a direct influence on all these variables and thus has an indirect influence on the IPA. Therefore, fostering a culture in the PE classroom that develops or promotes physical exercise identity will help achieve one of the most important objectives of the class: adherence to physical activity and sports practice not only during the school years but also throughout the lifetime. With this aim, different guidelines are proposed below to promote the development of the above variables during PE classes (see Table 6).

These proposed guidelines aim to give PE teachers some ideas to help them to achieve the big objective, namely, the physical activity adherence of their students. The use of new technologies is also important to promote adherence to physical activity. Gamification during this stage could be also a good strategy to keep the adolescents active, and even more important, to engage them in physical activity practice.

### Limitations and Future Lines of Research

Finally, it is necessary to highlight some limitations. The results in this paper are obtained from a specific population (secondary school and A-level students from one school in Spain) and therefore cannot be generalized to the whole population of secondary school students. Besides, the sample size of the research is limited, which might alter the results obtained in the SEM model. Therefore, in future research, it would be interesting to extend the sample to include secondary students from different institutions in Spain or other countries as well as to schools of different ownership types (public, private) to make comparisons. Moreover, this research analyzes the effect of age through a cross-sectional research design. Thus, future longitudinal studies should be developed to analyse the effect of adolescents’ age on their intentions to be physically active. In addition, although several guidelines have been established to promote the development of these variables through PE classes, they have not been widely implemented. It would also be interesting to carry out this same study but with a proposal for intervention, following the guidelines proposed and carrying out a quasi-experimental design to check whether the expected results are produced. Finally, the effect of gender in the predictive variables of the intention to be physically active has not been considered in the SEM model due to the limited sample size. Thus, future studies should consider the effect of gender and age together when analysing the predictive variables of the IPA with SEM.

## 5. Conclusions and Implications

Physical Education classes are an ideal setting to promote habits of physical activity and sports practice that last throughout a student’s life. PE teachers have to create attractive lessons to make students feel that this subject is something attractive and to encourage engagement in extracurricular physical activities. Although the number of hours is limited to achieve this ambitious goal, this is the ultimate goal that PE teachers must seek to achieve with their subject. In this way, they will be contributing socially to improving the quality of life of these adolescents and future adults. Therefore, quality physical education classes must be developed, so that the students can enjoy them. Moreover, in these lessons, they should be sensitized to the importance of physical sports practice and get the necessary tools to practice autonomously outside of lessons.

Finally, when Physical Education teachers design their classes should also consider the age of their students. The older their students are, the most important it is that they are aware of the importance of practicing physical activity regularly and that they perceived themselves as capable of developing this behaviour. At the end of Secondary School and A levels, students should have a positive attitude towards physical activity and be aware of its importance for their health. Besides, they should perceive that they can practice physical activity regularly. Thus, Physical Education teaches plays a vital role in promoting physical activity habits in the population.

## Figures and Tables

**Table 1:** Sample characteristics concerning sports habits.

	Yes (%)	No (%)
Extracurricular sport practice	83.10	16.90
Fathers‘ sport practice	63.30	36.70
Mothers’ sport practice	44	56
Friends’ sport practice	90	10
Physical education attractiveness	93.60	6.40

**Table 2:** Analysis of means, reliability (α), and correlation between variables with the intention of being physically active after completing secondary school.

	$\bar{X}$	SD	Range	V1	V2	V3	V4	V5	V6	V7
IPA	4.26	0.78	1–5	1						
ATB	4.16	0.71	1–5	0.34 ***	1					
PBC	4.46	0.79	1–5	0.41 ***	0.19 **	1				
SN	3.76	10.00	1–5	0.31 ***	0.21 **	0.25 ***	1			
PSC	3.61	0.76	1–5	0.50 ***	0.26 ***	0.24 ***	0.23 ***	1		
PEI	4.00	10.10	1–5	0.81 ***	0.41 ***	0.51 ***	0.39 ***	0.51 ***	1	
Age	16.36	0.62	16–19	−0.07	−0.04	−0.01	−0.05	−0.02	−0.09	1

Note: ** *p* ≤ 0.01 *** *p* ≤ 0.001. SD = Standard Deviation; IPA = Intention to be physically active after completing secondary school; ATB = Attitude towards the behaviour; PBC = Perceived behavioural control; SN = Subjective norm; PSC = Physical self-concept; PEI = Physical exercise identity.

**Table 3:** Means, Standard Deviation, and percentiles of 10,50, and 90 of the variables.

		IPA	ATB	PBC	SN	PSC	PEI	Age
Mean		4.30	4.17	4.48	3.77	3.62	4.02	15.63
Standard Deviation		0.73	0.70	0.77	1.00	0.75	1.06	1.12
Minimum		1.80	1.83	1.00	1.00	1.13	1.00	14
Maximum		5.00	5.00	5.00	5.00	5.00	5.00	19
Percentiles	10	3.20	3.33	2.00	2.25	2.63	2.20	14.00
	50	4.60	4.17	4.00	4.00	3.63	4.40	16.00
	90	5.00	5.00	5.00	5.00	4.63	5.00	17.00

Note: IPA = Intention to be physically active after completing secondary school; ATB = Attitude towards the behaviour; PBC = Perceived behavioural control; SN = Subjective norm; PSC = Physical self-concept; PEI = Physical exercise identity.

**Table 4:** Necessary conditions for high and low levels of IPA.

	IPA		~IPA	
	Consistency	Coverage	Consistency	Coverage
ATB	0.72	0.72	0.56	0.52
~ATB	0.50	0.54	0.67	0.69
PBC	**0.95**	**0.59**	0.83	0.49
~PBC	0.17	0.50	0.30	0.86
SN	0.68	0.70	0.53	0.53
~SN	0.54	0.55	0.70	0.68
PSC	0.55	0.85	0.37	0.56
~PSC	0.72	0.54	**0.90**	**0.66**
PEI	0.84	0.79	0.48	0.44
~PEI	0.40	0.44	0.77	0.82
Age	0.53	0.63	0.53	0.62
~Age	0.68	0.60	0.68	0.58
Gender	0.64	0.59	0.46	0.41
~Gender	0.36	0.41	0.54	0.53

Note: IPA = Intention to be physically active after completing secondary school; ATB = Attitude towards the behaviour; PBC = Perceived behavioural control; SN = Subjective norm; PSC = Physical self-concept; PEI = Physical exercise identity. Necessary conditions for IPA and ~IPA have been highlighted in bold.

**Table 5:** Sufficient conditions (intermediate solution) for high and low levels of IPA.

*Cut-Off* *Frequency*: 3	IPA *Cut-Off* *Consistency*: 0.80			~IPA *Cut-Off Consistency*: 0.81			
	1	2	3	1	2	3	4
ATB	●		●	●		○	
PBC	●	●	●	●			
SN							○
PSC							
PEI	●	●		○	○	○	
Age		○	○			○	○
Gender			●		○		○
Consistency	0.80	0.82	0.84	0.88	0.86	0.89	0.83
Raw coverage	0.61	0.59	0.31	0.47	0.42	0.41	0.26
Unique coverage	0.14	0.13	0.02	0.06	0.10	0.02	0.03
Total solution consistency	0.78			0.85			
Total solution coverage	0.76			0.70			

Note: ● = presence of condition, ○ = absence of condition; Expected vector for intrapreneurial intentions: 1.1.1.1.1.1-0. (0: absent; 1: present); Expected vector for ~ intrapreneurial intentions: 0.0.0.0.0.1-0 using the format of Fiss [64]. IPA = Intention to be physically active after completing secondary school; ATB = Attitude towards the behaviour; PBC = Perceived behavioural control; SN = Subjective norm; PSC = Physical self-concept; PEI = Physical exercise identity.

**Table 6:** Guidelines to promote physical self-concept, physical exercise identity, attitude towards behaviour, and perceived behavioural control in PE classes.

Variables	Guidelines for PE Classes	
Physical exercise identity and physical self-concept	- Use the step counter pedometer application to encourage students to walk instead of going by car or bus whenever they can and analyse the number of steps they take during the week. - Encourage students to participate in a sporting event or activity outside the classroom by giving an extra point (0.10 per activity). - Make a diary of sports habits and reward students throughout the term (recognition of the most active student of the week).	(1) Teach students how to measure their basic skills through different tests at the beginning of the first term. (2) Once these skills have been measured, propose to the students that they set some improvement objectives for the end of the term. (3) Give the students a dossier with different activities, so that they can carry out their sessions during PE classes and outside them. (4) Sign a commitment document with the students to exercise at least one more hour outside the classroom, in addition to the two hours of physical education per week. (5) The teacher supervises the sessions that the students present for the next day to avoid injuries and health risks. (6) Students re-measure their basic abilities to learn whether they have achieved the objectives they previously set themselves.
Attitude towards behaviour	- Bring professional sportsmen and women to Physical Education classes, so that they can tell them about their experiences and carry out some dynamic activities. - Show students a wide variety of sports during PE classes to arouse interest in any of the sports modalities.	
Perceived behavioural control	- Carry out activities of greater interest to the students in the class and allow them to work autonomously. For example, have students conduct a warm-up or session. - Show them how to use free apps to monitor their physical exercise.	
Subjective norm	- Organise informative sessions and talks with the students’ families about the benefits of practicing physical activity. - Organise activities and days of physical and sporting practice in which students participate together with their parents and classmates/friends	
Age	- The younger the adolescents are (e.g., 1st, 2nd year of secondary school), the more PE classes should focus on working on the control of vigorous sports practice regularly and the development of their physical exercise identity. It is necessary to improve the perception of both variables (see guidelines on perceived behavioural control and physical exercise identity). - Specifically, in the case of male adolescents, is quite important that PE lessons develop a positive perception of the importance of regular physical activity practice and on working on the control of vigorous sports practice regularly (see guidelines on perceived behavioural control and attitude towards behaviour). - In the case of young female adolescents, if very important that their close environment (family, friends…) perceive them as capable of practicing physical activity regularly. Thus, it is very important that PE teachers involve the immediate environment in activities related to physical activity practice (see guidelines for the subjective norm).	

## Data Availability

Data available on request.
